# Crop Pest Recognition in Real Agricultural Environment Using Convolutional Neural Networks by a Parallel Attention Mechanism

**DOI:** 10.3389/fpls.2022.839572

**Published:** 2022-02-21

**Authors:** Shengyi Zhao, Jizhan Liu, Zongchun Bai, Chunhua Hu, Yujie Jin

**Affiliations:** ^1^Key Laboratory of Modern Agricultural Equipment and Technology, Jiangsu University, Zhenjiang, China; ^2^Research Institute of Agricultural Facilities and Equipment, Jiangsu Academy of Agricultural Sciences, Nanjing, China; ^3^College of Information Science and Technology, Nanjing Forestry University, Nanjing, China

**Keywords:** crop, pest recognition, deep learning, convolution neural network, attention mechanism

## Abstract

Crop pests are a major agricultural problem worldwide because the severity and extent of their occurrence threaten crop yield. However, traditional pest image segmentation methods are limited, ineffective and time-consuming, which causes difficulty in their promotion and application. Deep learning methods have become the main methods to address the technical challenges related to pest recognition. We propose an improved deep convolution neural network to better recognize crop pests in a real agricultural environment. The proposed network includes parallel attention mechanism module and residual blocks, and it has significant advantages in terms of accuracy and real-time performance compared with other models. Extensive comparative experiment results show that the proposed model achieves up to 98.17% accuracy for crop pest images. Moreover, the proposed method also achieves a better performance on the other public dataset. This study has the potential to be applied in real-world applications and further motivate research on pest recognition.

## Introduction

Agriculture is an important basic industry worldwide, and pests can cause huge losses to crop production in every country (Santangelo, 2018). According to research, nearly half of global crop production will be impacted to varying degrees due to pests every year, which seriously affects the regional economy and people’s daily lives (King, 2017). Pest detection has become an important task for the development of agricultural precision because pests have a wide distribution, cause great damage, and reproduce quickly (Wang et al., 2020). Traditional pest detection methods mainly include manual inspection and light trapping, but these methods need manual intervention and experience problems related to insufficient automation and intelligence, such as a large workload, low efficiency, and poor real-time performance (Lim et al., 2018). Due to the diversity of pests, manual identification relies on a large amount of expert knowledge, and it is difficult to obtain accurate and timely information on the number and species of pests in orchards, so it is difficult to widely implement (Li Y. et al., 2020). The automatic recognition of pests can provide a better growth environment for crops and increase the level of agricultural production.

With the rapid development of computer vision and pattern recognition technology, machine learning and deep learning have become the main research directions of agricultural pest detection (Albanese et al., 2021; Liu and Wang, 2021). For example, Fina et al. (2013) proposed a pest identification method using k-means clustering segmentation, but it takes a long time to label features manually in the case of a large dataset. Zhong et al. (2018) used a Prewitt operator and Canny edge detection algorithms to extract the morphological features of pests. Then, a support vector machine (SVM) was used to automatically recognize whiteflies and thrips, and the experimental results showed that the recognition rate was nearly 90%. Liu T. et al. (2016) proposed a method for the detection of wheat aphids based on genetic algorithms, which can accurately identify and count in the complex environment of the field. Yaakob and Jain (2012) used six invariant matrices to extract the shape features of pests, then combined the ARTMAP neural network algorithm and achieved an 85% recognition accuracy in a specific background. Barbedo (2014) developed a soybean whitefly monitoring system based on digital image processing, which can realize the automatic identification and counting of whiteflies and greatly improves work efficiency compared with manual inspection. Although the traditional machine learning recognition algorithm has achieved better results when the number of crop pest species is small, when there are many kinds of pests and the input parameters are limited, the machine learning method has difficulty effectively extracting key feature information, resulting in poor performance of the model robustness (Roy and Bhaduri, 2021).

Deep learning is an autonomous machine learning method that uses multilevel neural networks, and computers can automatically extract key features from a large number of images (Brahimi et al., 2017). Saleh et al. (2021) has demonstrated convolutional neural network (CNN) is a high performance deep learning network, and the CNN has the best performance compared to multiple models (DT, RF, SVM, NB, LR, KNN, RNN, and LSTM). CNN abandons complex preprocessing and feature extraction operations, and uses an end-to-end architecture that effectively combines global and local features and greatly simplify the recognition process. Thus, CNNs have been widely used in crop information recognition for real agricultural environments, and the automatic recognition of pests combined with CNNs is conducive to improving the accuracy of detection and reducing labor costs (Cheng et al., 2017).

Many studies have been carried out on the use of deep learning technology for crop key informations detection to provide accurate information for subsequent spray management, effectively improving the survival rate and yield of vegetables, fruits and field crops. A model of classification of tomato leaf diseases and pests with 89% accuracy was designed (Shijie et al., 2017), but this method can be applied in simple background pest classification and is impossible to integrate into practical applications. Chen et al. (2019) improved the residual network structure, added a high-resolution convolutional layer and the corresponding number of channels, and the accuracy of pest identification reached 91.5%. Wang et al. (2020) fused pest context information into a CNN, which improved the accuracy of pest detection and recognition in complex environments. Liu et al. (2019) proposed an effective multiscale data enhancement method for pest images. This method combines different scale image enhancements into the recognition model, which solves the problem that the traditional single image scale algorithm cannot be applied to the detection and recognition of small target pests. A method using CNN architecture for fruit fly recognition was proposed and achieved an accuracy of 95.68% (Leonardo et al., 2018). Generative Adversarial Networks (GAN) were applied to extend the dataset, and the extended dataset was fed into a pre-trained CNN model, which achieved an accuracy of 92% for plant disease classification (Gandhi et al., 2018). Dawei et al. (2019) designed a diagnostic system based on transfer learning for pest detection, and this approach to train and test 10 types of pests and achieves an average accuracy of 93.84%.Chen et al. (2021) proposed to classify tea pests by fine-tuning the VGG-16 network, and the results showed that the classification has accuracy up to 97.75%.

In recent years, due to the characteristic of extracting discriminative features of the area of interest, the attention mechanism has begun to be widely used in machine translation, generative adversarial and so on (Dong et al., 2019; Xiang et al., 2020). Researchers used the attention mechanism to quickly scan a global image to obtain the region of interest. However, it is still in the exploratory stage in the field of crop pest recognition. Liu et al. (2019) proposed a pest identification method based on CNN technology. This method combined the channel attention mechanism into the CNN. Through experiments on 16 types of field pests, the average accuracy reached 75.46%, and the accuracy was significantly improved. Guo et al. (2020) designed a self-attention mechanism and incorporated it into the CNN structure, which achieved the optimal F1-scores of 93.21% for 11 types of crop diseases and pests. Zhang and Liu (2021) proposed a method based on DenseNet and an attention mechanism, and the model could identify 7 types of navel orange diseases and pests on the test set with 96.90% accuracy. The results in this study are compared with on other studies as summarized in Table 1.

**TABLE 1:** Summary of the comparison of the existing work.

Paper	Object	Model	Types	Accuracy
Shijie et al. (2017)	Diseases and pests	VGG-16 + Transfer learning	9	89.00%
Chen et al. (2019)	Pests	ResNet + Block-cg	38	91.50%
Wang et al. (2020)	Pests	ResNet-50	3	72.30%
Liu et al. (2019)	Pests	CNN + Attention	16	75.46%
Leonardo et al. (2018)	Pests	SVM + VGG-16	10	95.68%
Dawei et al. (2019)	Pests	AlexNet + Transfer learning	10	93.84%
Chen et al. (2021)	Pests	VGGNet-16	14	97.75%
Guo et al. (2020)	Diseases and pests	CNN + Self-attention	11	92.78%
Zhang and Liu (2021)	Diseases and pests	DenseNet + Attention	7	96.90%
Our model	Pests	ResNet-50 + Parallel-attention	10	98.17%

By analyzing current work, deep learning methods have been proven to significantly improve pest recognition performance, providing a reference for the recognition of crop pests. However, these studies mostly focus on the improvement and optimization of the diseases and pests recognition model. On the application of deep learning models, Alsamhi et al. (2021) combination of neural networks and IoT devices plays a vital role in improving feedback control efficiency with automatic operation and reductions of fertilizer and pesticides consumption. Agricultural UAVs are a modern agricultural technology with remarkable efficiency in quickly identifying and locating areas of outbreaks of pests and diseases through aerial imaging. And combining UAVs with high-performance IoT sensors enables efficient tasks such as remote crop growth monitoring, soil moisture monitoring, and water quality monitoring (Almalki et al., 2021). Meanwhile, UGVs have also been widely used for crop planting monitoring, and by deploying a crop information detection model on the controller, it has been achieved soil moisture, pH, fertility monitoring and climate conditions monitoring, crop plant diseases and insect pests monitoring, growth and yield monitoring, etc. (Jin et al., 2021).

In the recognition task, pest pixels only occupy a small part of the whole image, and the attention mechanism can improve the learning of important feature channels of pests. The proposed model added a parallel attention module with a CNN structure to automatically extract pest feature information from a real agricultural environment. Feature extraction is focused on the pest feature channel, and invalid feature channel information is eliminated. Thus, the proposed model in this paper can automatically accurately recognize ten types of crop pests.

The main contributions of this paper are summarized as follows:

(1) To meet the recognition requirements of crop pests, this paper collects 10 types of pest images in a real agricultural environment. Thus, data enhancement improves the robustness and accuracy of the model performance in the detection task.
(2) This paper proposes an improved CNN model for the recognition of crop pests. Based on the original residual structure, spatial attention is combined with channel attention to obtain a parallel attention mechanism module. The parallel attention module is deeply integrated into the ResNet-50 network model.
(3) The attention module can establish a multidimensional dependency relationship of the extracted crop pest feature map, is lightweight and can be easily added into the network. Using this method, we achieved highly accurate recognition of crop pests in complex agricultural environments.

This paper is divided into five sections. The model improvement methods are shown in section “The Proposed Approach.” Section “Experiment” shows the dataset collection and experiment setup. The performance of the deep learning method is discussed in section “Experimental Results and Discussion,” and conclusions and future work are described in section “Conclusion.”

## The Proposed Approach

### Spatial/Channelwise Attention Mechanism

#### Spatial Attention Mechanism

Researchers have proposed a variety of attention mechanisms and applied them to the training tasks of CNN models. At the cost of smaller calculations and parameters, the network performance can be greatly improved (Fukui et al., 2019). The attention mechanism mainly includes the channel attention mechanism and spatial attention mechanism. The spatial attention mainly extracts important regions in the feature and judges the importance of the corresponding feature by the dependence between different positions in the feature. The corresponding weight parameters are assigned to improve the feature expression of the key area. Therefore, spatial attention enables the network to better evaluate the effect of each feature position during the classification feature extraction process and further enhances the modeling ability of the network.

**FIGURE 1:**
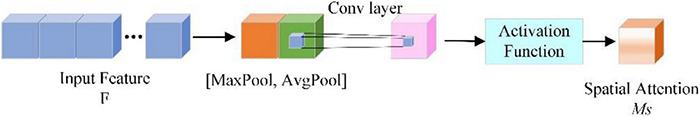
The structure of the spatial attention mechanism.

As shown in Figure 1, average pooling and maximum pooling operations are performed on the input feature map *F*, and information is gathered separately into two different feature maps and used convolutional layers are applied to generate spatial attention maps *Ms.* Then, feature fusion is realized through a 7 × 7 convolution operation, and the sigmoid activation function is used to generate a weight map and superimpose it on the original input feature map. Finally, the features of the target pixel area are enhanced.

#### Channel Attention Mechanism

Channel attention mainly performs correlation modeling on the feature maps of different channels, adaptively obtains the importance of each feature channel through back-propagation parameter learning, and assigns different weight coefficients to each channel.

**FIGURE 2:**
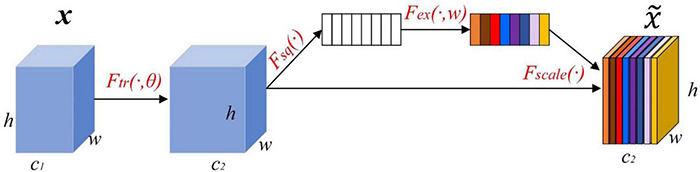
The structure of the channel attention mechanism.

SENet is one of the classic channel attention modules, as shown in Figure 2. Hu et al. (2018) mentioned it in a CVPR ImageNet Workshop speech. The weights of different channels are trained through the cost function, and the weight coefficients of each feature channel are automatically obtained. Then, according to the size of the weight coefficient of each feature channel, the effective feature channel is enhanced, and the invalid feature channel is suppressed.

### Parallel Attention Mechanism Design

**FIGURE 3:**
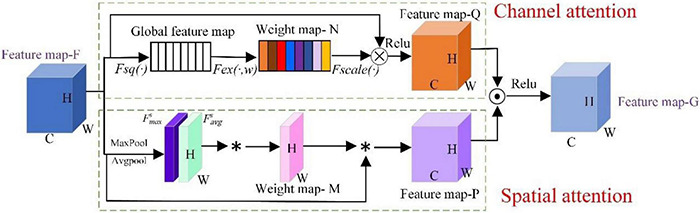
The structure of the parallel attention mechanism.

Based on pest recognition, we know that features from the spatial attention module are highlighted in pest regions from the perspective of spatial position, while features from the channelwise attention module are highlighted from the perspective of channels, which carry more important information at the channel level. It is necessary to combine multi-attention features together to obtain enhanced attention features. Therefore, this paper proposed a parallel attention mechanism, namely, PCSA, that effectively combines the spatial attention module and the channel attention module in series as that used for pest recognition. In Figure 3, the PCSA consists of three parts: channel attention, spatial attention and feature map fusion. It can be directly applied to existing network architectures.

(1) The channel attention mainly redistributes the channel weights in the feature map through one-dimensional convolution, increases the weight of pest-related channels and reduces the weight of the remaining channels. First, the global average pooling calculation is performed on the feature map with input size *C* × *H* × *W* through the squeeze operation (*F*_*sq*_) to obtain a *1* × *1* × *C* feature vector and it is input into the two fully connected layers. The *ReLU* activation function is used between the two fully connected layers, generated feature maps are first downscaled by FC-1 and then upscaled by FC-2, and the feature channel dimensions of the input and output are the same. The squeeze process can be expressed as follows:


$$F_{\mathrm{sq}}\left(u_{c}\right)=\frac{1}{H\times W}\sum_{i=1}^{H}\sum_{j=1}^{W}u_{c}\left(i,j\right)$$

Where *u_*c*_(i,j)* is the element in row *i* and column *j* of the input data. Then, the input feature map *F* generates a *1* × *1* × *C* global feature map. In the excitation operation (*F*_*ex*_), the sigmoid activation function is used to calculate the weight of each feature channel, which is the core of the entire channel attention module. These weights are allocated to the input feature maps. The excitation process can be expressed as follows:


$$F_{\mathrm{ex}}\left(z,W\right)=\sigma \left(g\left(z,W\right)\right)=\sigma \left(W_{2}\sigma \left(W_{1}z\right)\right)$$

Where σ is the ReLU function, *z* is the result of the compression process. The parameter *W*_1_ reduces the dimension of channels to *1/r* of the original in the FC-1, restore the dimension of channels to the original dimension of channels with parameter *W*_2_ in the FC-2, and *W*_1_ and *W*_2_ are inverse relationships. *W_1_∈*$R^{{\frac{C}{r}}^{*}C}$ and *W_2_∈*$R^{C^{*}\frac{C}{r}}$ are the downgrading and upgrading parameters of the FC-1 and FC-2, *r* is the scaling parameter to balance model performance and computational complexity. Finally, the output feature channel weight vector is multiplied by the original input feature map through the scale operation (*F*_*scale*_) to complete the original feature calibration in the channel dimension. Therefore, the extracted features have stronger directivity and improved classification performance. The scale process can be expressed as follows:


$$F_{\mathrm{scale}}\left(u_{c},s_{c}\right)=u_{c}⊙s_{c}$$

Where *F_*scale*_(u_*c*_, s_*c*_)* refers to the channelwise multiplication between the scalar *s*_*c*_ and the feature map *u_*c*_∈R^H×W^*.

(2) Spatial attention performs average pooling and maximum pooling operations on the feature map *F* in the channel dimension and generates two single-channel feature maps $F_{avg}^{s}$ and $F_{max}^{s}$. Then, the $F_{avg}^{s}$ and $F_{max}^{s}$ feature maps are combined to generate a weight map *M*, and the feature map *F* is weighted by the weight map *M* to generate a feature map *P*. Finally, in the feature map *P*, the areas related to the pests are given higher weights, while the other areas have lower weights. The calculation process of the spatial attention module can be expressed as follows:


$$M_{s}=\left(\left[Avgpool\left(F\right)⊗maxpool\left(F\right)\right]\right)=\sigma \left(\left[F_{avg}^{s}⊗F_{max}^{s}\right]\right)$$

Where σ is the ReLU function, *s* is the 2D feature maps and is the dot product of position data corresponding to $F_{avg}^{s}$ and $F_{max}^{s}$ feature maps.

(3) The feature map *Q* is dot-producted with the feature map *P* and the feature map *G* is obtained using the ReLu activation function. The feature map *G* combines the weight distribution of the channel dimension and the weight distribution of the spatial dimension, thereby obtaining complementary key features, which can highlight the pest feature area and suppress various interferences, so that the model can identify pests more accurately.

### Crop Pest Recognition Model of ResNet-50 Fused to PSCA

Feature extraction is the key part of deep learning models, and the convolutional layer of the CNN has powerful feature extraction capabilities. Recently, AlexNet, VGGNet and GoogLeNet have been widely used in face recognition, disease diagnosis, text classification and other tasks and have achieved good results (Ballester and Araujo, 2016). However, these CNNs increase the feature extraction ability by adding to the number of network layers, which will increase the number of model parameters and the computational cost (Tang et al., 2020b). More seriously, it will cause the problems of network redundancy, gradient explosion and disappearance.

The residual network proposed by He et al. (2016) won the championship in the 2015 ImageNet large-scale visual recognition competition. The residual block in the model can avoid the problem of network degradation caused by the deepening of the number of network layers. Compared with AlexNet, VGGNet, and GoogLeNet, ResNet has less computation and higher performance. Compared with ResNet-101 and ResNet-18, ResNet-50 has the advantages of higher accuracy, fewer parameters and faster speed (Li X. et al., 2020). Thus, this study chose ResNet-50 as the feature extraction network.

**FIGURE 4:**
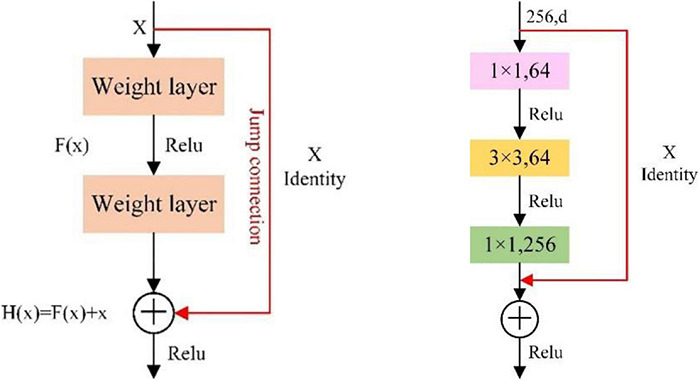
The residual block **(left)** and the bottleneck structure **(right)**.

**FIGURE 5:**
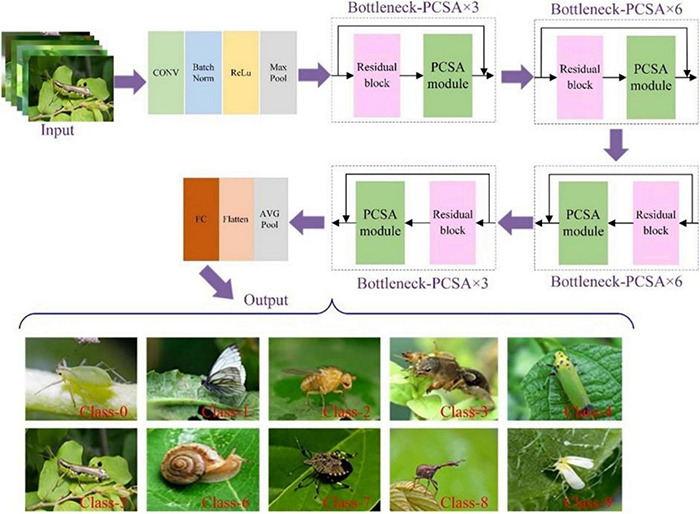
The structure of the crop pest recognition model.

In Figure 4, identity mapping uses the jump connection method to directly add feature *X* that the network originally wants to learn from the shortcut branch and feature *F_(*X)*_* learned from the weighted layer through the *ReLU* activation function. The bottleneck structure in the ResNet network can effectively reduce the network parameters and computational complexity. The bottleneck structure is composed of two *1* × *1* convolutional layers and one *3* × *3* convolutional layer. The input feature vector is reduced from 256 dimensions to 64 dimensions through a *1* × *1* convolution, a *3* × *3* convolutional layer is used to learn features, and the feature vector is restored to 256 dimensions through a *1* × *1* convolutional layer. Finally, the identity map and output are added through the *ReLU* activation function. In this paper, a PCSA is added to the original model structure of ResNet-50 to obtain the ResNet-50-PCSA model. The network architectures of the improved ResNet-50 are depicted in Figure 5.

The model mainly includes four stage processes, and each stage is composed of a residual module. The proposed model embeds the PCSA module after the residual module and constitutes 4 bottleneck-PCSA modules, the numbers of which are 3, 6, 6, and 3. The size of the convolution kernels of bottleneck-PCSA is the same. The main difference between models is the number of convolution kernels and the output dimensions of the fully connected layer in the PCSA module. The crop pest images are input into the ResNet-50-PCSA network structure, first through the convolutional layer, BN layer, activation layer and max pool. Then, the pest feature map was obtained through 4 bottleneck-PCSA modules. Finally, the obtained feature map is calculated by AVG pooling, and the number of output feature layers is changed from multidimensional to one-dimensional through the flattened layer and output through the fully connected layer. When deepening the number of network layers, if the internal features of the network have reached the optimal level in a certain layer, the subsequent superimposed network layers will not change the features.

The above is the complete structure and operation process of the ResNet-50-PCSA model. The PCSA subnetwork structure is embedded in ResNet-50. The combination of the feature channel recalibration strategy and residual network can effectively improve network performance and thus does not need to greatly increase the computational cost. Through feature refinement, the learning ability of complex pest features is enhanced.

## Experiment

### Dataset Acquisition

The development of deep learning in recent years has proven that the detection and classification tasks of target objects can be effectively achieved under high-quality and large-size datasets (Liu W. et al., 2016). For crop pests, their active time and distribution law are related to various environmental factors, such as climate and season, and it is difficult to obtain large images. Therefore, it is not feasible to obtain a large number of pest images through the process of collecting and shooting. This paper makes use of abundant internet resources to compensate for this deficiency and enriches the content of image data by open-source dataset and web crawler methods.

**FIGURE 6:**
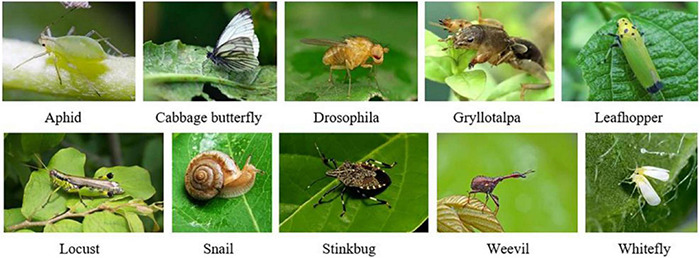
Sample images for 10 common pest classes.

In this paper, we selected 10 common classes of crop pests, namely, Aphid, Cabbage butterfly, Drosophila, Gryllotalpa, Leafhopper, Locust, Snail, Stinkbug, Weevil, and Whitefly, as shown in Figure 6. Because these pests are prone to exist all over the world, they reproduce very quickly and spread widely (Dawei et al., 2019). They mainly feed on the leaves, stems and fruits of crops. If they lay eggs on crops, they are difficult to handle and will cause huge losses in crop yields. Therefore, effective detection and timely control of these 10 types of pests have great significance.

Most of the images in the pest dataset in this paper are collected from the internet, and a few are from the open-source dataset. The web crawler keywords for each type of pest were divided into Chinese and English. Multithreaded collection of the images of each type of pest is completed using three major internet search engines: Google, Bing and Baidu. The open source dataset mainly comes from Kaggle^1^ and Forestry^2^. Although the key information is defined in the collection process, there are still many non-pest images and redundant data. Several agricultural technology experts judged and classified the collected images and removed incorrect pest types and poor-quality images. The size of all images was unified by means of image normalization (*224* × *224*), and the format was JPG. Overall, there were more than 400 images of each type of pest, and the number of snails and locusts exceeded 800 images.

#### Data Augmentation

Data augmentation is an important data processing technology in deep learning. It can effectively increase the amount and diversity of training data and improve the generalization ability and robustness of the model (Shorten and Khoshgoftaar, 2019). Data enhancement is divided into online enhancement and offline enhancement; online enhancement is suitable for large datasets, and after the model obtains batch data, it can be enhanced by rotation, translation and folding (Tang et al., 2020a). Offline enhancement directly processes images and is suitable for small datasets. Therefore, this paper used offline augmentation techniques and enhanced images in combination with OpenCV under the PyTorch framework.

a) Spin: Randomly rotating the picture by 0°, 90°, 180°, and 270° will not change the relative position of the pest pixels, simulating the randomness of the shooting angle under natural conditions.
b) Zoom: The images are reduced according to a certain ratio, which helps to identify pests on multiple scales. For the scaled image, the resolution of the image is expanded to 224 × 224 pixels by filling in fixed color pixels.
c) Gaussian noise is added to the image to simulate the interference information in the natural environment.
d) Color jitter: Changed the image brightness and contrast to simulate the image difference generated by the change of light intensity in the environment of crop growing. The color jitter can be expressed as follows:

$$g\left(i,j\right)=b^{*}f\left(i,j\right)+a;a\in \left[a_{1},a_{2}\right]$$

where *a* is the image contrast, *b* is the image brightness, *g(i,j)* is the output image, *f(i,j)* is the input image, *a*_1_ is the lowest brightness factor in the field and *a*_2_ is the highest brightness factor in the field.

**FIGURE 7:**
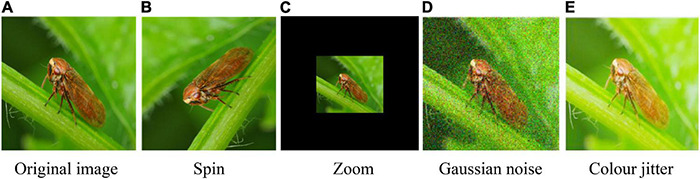
Samples for data augmentation. **(A)** Original image, **(B)** spin, **(C)** zoom, **(D)** Gaussian noise, and **(E)** color jitter.

Samples of the data enhancement is shown in Figure 7. By using these image offline augmentation techniques, the number of datasets is expanded four times. The total number of original images was 5,245; after data augmentation, the number of images increased to 26,225. The training set and validation set are divided into 8:2 ratios, and detailed information on the dataset are shown in Table 2. For the model testing, we collected 150 real images of each pest and formed a testset. In the end, the testset contained 1,500 images.

**TABLE 2:** Crop pest dataset detail information.

Pest	Class	Origin images	Augmentation images	Trainset	Validation set
Aphid	0	415	2,075	1,660	415
Cabbage butterfly	1	430	2,150	1,720	430
Drosophila	2	440	2,200	1,760	440
Gryllotalpa	3	485	2,425	1,940	485
Leafhopper	4	455	2,275	1,820	455
Locust	5	820	4,100	3,280	820
Snail	6	850	4,250	3,400	850
Stinkbug	7	420	2,100	1,680	420
Weevil	8	480	2,400	1,920	480
Whitefly	9	450	2,250	1,800	450
**Total**		5,245	26,225	20,980	5,245

### Experiment Setup

In this study, the weight parameters of the pretrained ResNet-50 model on ImageNet are used for transfer learning to accelerate the convergence speed of the model. The collected dataset contains 10 kinds of pests, so the output layer must be changed from 1,000 (ImageNet pretrained ResNet-50) to 10. The operating platform for this experiment is a Dell T7920 graphics workstation, the operating environment is Windows 10, the CPU is Intel Xeon Gold 6248R, and the GPU is NVIDIA Quadro RTX 5000. The training environment is created by Anaconda3, and the environment configuration is Python 3.6 and PyTorch 1.8.0, torchvision 0.7.0 artificial neural network library. The model parameters were selected as follows: the initial learning rate set to 0.001, a weight decay of 0.00001 and momentum factor is 0.1. Set 100 epochs, after 2 epochs, the model performance does not improve and the learning rate will decrease after that. At the same time, the CUDA 10.2 deep neural network acceleration library is used. The experiment uses a stochastic gradient descent with momentum (SGDM), updates the parameters and optimizes the training process. The parameter update can be expressed as follows:


$$\theta_{i+1}=\theta_{i}-\alpha \Delta L_{R}\left(\theta_{i}\right)+m\left(\theta_{i}-\theta_{i-1}\right)$$

where *i* is the number of iterations, θ is the network parameters, Δ*L*_*R*_(θ_*i*_) is the loss function gradient, *m* is the momentum and α is the learning rate. Meanwhile, before the training and validation of each epoch, the data was randomly shuffled. After each training, the validation set is tested, and the model is saved. Finally, the model with the highest accuracy is selected.

### Model Evaluation Index

When evaluating the performance of a model, Precision (*P*), Recall (*R*), F_1_ Score (*F*_1_) and Detection speed (*T*_*a*_) are usually selected as evaluation indices.


$$P=\frac{T_{P}}{T_{P}+F_{P}}$$

$$R=\frac{T_{P}}{T_{P}+F_{N}}$$

$$F_{1}=2\times \frac{P\times R}{P+R}$$

Where *T*_*P*_ (true positive) is the number of positive samples predicted as positive samples, *F*_*P*_ (false-positive) is the number of negative samples considered to be positive samples, and *F*_*N*_ (false negative) is the number of positive samples considered to be negative samples.


$$T_{a}=\frac{T}{N}$$

Where *T* is the total detection time for the validation set, and *N* is the total number for the validation set.

## Experimental Results and Discussion

### Comparison of the Performance of Various Models

To evaluate the performance, the proposed network is compared with several famous CNN networks, such as VGG-19, AlexNet, ResNet-101 and GoogLeNet. These models were configured to use the same optimizer (SGDM), classifier (softmax) and learning rate (0.0001).

**FIGURE 8:**
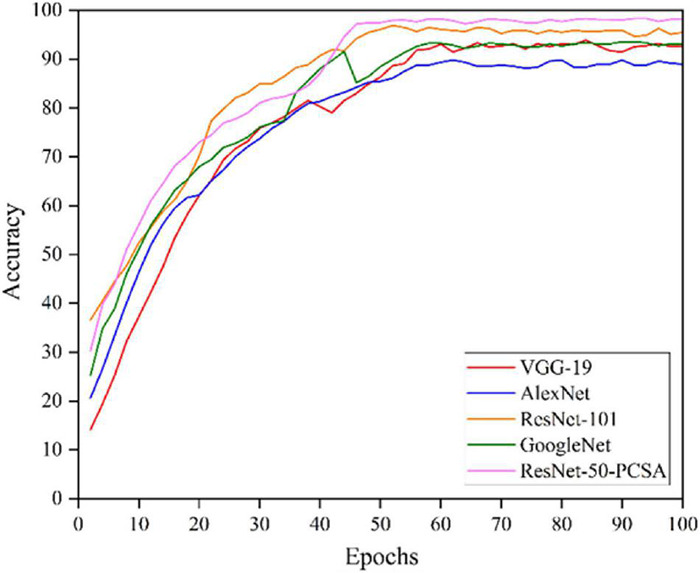
The training accuracy curves.

The comparison of various CNN model training curves is shown in Figure 8. The training iteration epochs are plotted on the x-axis, and the accuracy is plotted on the y-axis. The ResNet-50-PCSA model proposed in this paper has the highest accuracy, and except for the AlexNet model, the accuracy of the other models exceeds 90% because AlexNet is not deep enough compared to other models, and the amount of feature information extracted by the network is less. Meanwhile, the ResNet-50-PCSA model converges fastest, and the model begins to converge after approximately 45 epochs. The VGG-19, AlexNet, ResNet-101, and GoogLeNet models have larger fluctuations after convergence, and the ResNet-50-PCSA model converges with the smallest fluctuation range, reflecting good stability.

The detailed evaluation results of different models on crop pests are obtained in Table 3. Under the same experimental conditions, the ResNet-50-PCSA model proposed in this paper has the highest precision, recall and F_1_ score. The proposed model also has the highest average accuracy, with an accuracy reaching 98.17%. Compared with the VGG-19, AlexNet, GoogLeNet and ResNet-101 models, the average accuracy is 5.55, 9.21, 4.82, and 3.69% higher, respectively, and the proposed model is significantly ahead of the other CNN networks. The ResNet-50-PCSA model has the fastest recognition speed, and the average recognition time for a single pest image is only 32.29 ms. Compared with the second-ranked AlexNet model, the time is reduced by 1.08 ms, which meets the needs of real-time recognition of crop pests. Considering that the model will be deployed to the inspection robot system, VGG-19, AlexNet and ResNet-101 have a large model size, which cannot guarantee real-time detection task requirements. Moreover, the size of the ResNet-50-PCSA model is 91 Mb, which is 46 Mb larger than GoogLeNet. However, ResNet-50-PCSA also meets the requirements of lightweight deployment, and the accuracy is higher than that of GoogLeNet. Synthesizing the above analysis, the proposed model achieves the best performance in terms of accuracy and speed.

**TABLE 3:** The evaluation results.

Model	Input	*P*	*R*	F_1_	T_*a*_ (ms)	Accuracy (%)	Size (Mb)
VGG-19	224	0.9137	0.9130	0.9133	41.81	92.62	482
AlexNet	224	0.8905	0.8891	0.8898	33.37	88.96	227
GoogLeNet	224	0.9331	0.9324	0.9327	33.64	93.35	45
ResNet-101	224	0.9537	0.9548	0.9542	39.05	94.48	167
ResNet-50-PCSA	224	0.9798	0.9816	0.9807	32.29	98.17	91

### Effectiveness of PCSA Module

To prove the effect of adding a parallel attention mechanism on the performance of the original model, keeping the experimental conditions and parameters consistent, a comparison experiment of the performance of the ResNet-50-PCSA and ResNet-50 models was carried out. The results of the comparative experiment of the proposed model and the ResNet-50 model without a parallel attention mechanism on crop pests are shown in Table 4.

**TABLE 4:** The results of ResNet-50-PCSA compared with ResNet-50.

Model	Input	*P*	*R*	F_1_	T_*a*_ (ms)	Accuracy (%)	Size (Mb)
ResNet-50	224	0.9386	0.9391	0.9388	31.36	92.41	78
ResNet-50-PCSA	224	0.9798	0.9816	0.9807	32.29	98.17	91

It can be seen from Table 4 that the results of the model are improved after adding the parallel attention mechanism. The accuracy of the model is increased by 5.76%, and the precision, recall and F1 score are all higher than those of the original ResNet-50 model. The proposed model can retain more image details due to important feature reuse. However, the ResNet-50-PCSA model average detection time of a single pest image is increased by 0.93 ms, and the model size is increased by 13 Mb. This explains why adding the parallel attention mechanism can slightly increase the computational complexity and complexity of the model.

To further verify the effectiveness of the parallel attention mechanism proposed in this paper, we selected two widely used attention mechanisms as comparative experiments: SENet (Hu et al., 2018) and CBAM (Woo et al., 2018). The CBAM is composed of a serial structure of channel attention and spatial attention; it first learns the key features through the channel attention module and then uses the spatial attention module to learn the location of the key features.

The comparison results of the PCSA module with SENet and CBAM are shown in Table 5. In the recognition accuracy of the model, the ResNet-50-PCSA is 3.21 and 2.12% higher than ResNet-50-SENet and ResNet-50-CBAM, respectively. In terms of the average inspection time and model size, the ResNet-50-PCSA is slightly insufficient. ResNet-50-SENet has the fastest recognition speed and smallest model size. The average detection time is only 1.67 ms faster than ResNet-50-PCSA, but the recognition accuracy is significantly lower than ResNet-50-PCSA. The recognition speed of ResNet-50-PCSA still meets actual application requirements. At the same time, the model size of ResNet-50-PCSA is 19 and 5 Mb larger than ResNet-50-SENet and ResNet-50-CBAM, respectively, but it also confirms the requirements of lightweight deployment in machine control panels. Synthesizing the above analysis, the results show that the proposed parallel attention mechanism is effective.

**TABLE 5:** The results of PCSA compared with SENet and CBAM.

Model	Input	*P*	*R*	F_1_	T_*a*_ (ms)	Accuracy (%)	Size (Mb)
ResNet-50-SENet	224	0.9495	0.9496	0.9495	30.62	94.96	72
ResNet-50-CBAM	224	0.9601	0.9603	0.9602	31.98	96.05	86
ResNet-50-PCSA	224	0.9798	0.9816	0.9807	32.29	98.17	91

### Crop Pest Classification Results

Table 6 shows the ResNet-50-PCSA model accuracy of each pest on the validation set. The indices of 10 classes of pests are represented as follows: 0. Aphid, 1. Cabbage butterfly, 2. Drosophila, 3. Gryllotalpa, 4. Leafhopper, 5. Locust, 6. Snail, 7. Stinkbug, 8. Weevil, 9. Whitefly.

**TABLE 6:** Accuracy for crop pest recognition with 10 classes.

Classes	0	1	2	3	4	5	6	7	8	9
Accuracy (%)	97.08	98.37	97.15	99.34	97.53	98.52	99.15	98.43	98.01	97.10
**Average accuracy (%)**	**98.17**									

The result suggests that the model correctly recognizes 10 classes of pests with an average accuracy of 98.17%. The model recognition accuracy for aphid, Drosophila, leafhopper, and whitefly is low, but the accuracy also exceeds 97%. The reason is that the color features of aphids and leafhoppers are similar to those of crop leaves, and Drosophila and whiteflies are smaller in size and occupy only a few pixels in the whole image. Furthermore, the model exceeded 99% accuracy on 2 classes of pests (Gryllotalpa, and snail), while the other 4 classes of pests had accuracies between 98.01 and 98.52%.

**FIGURE 9:**
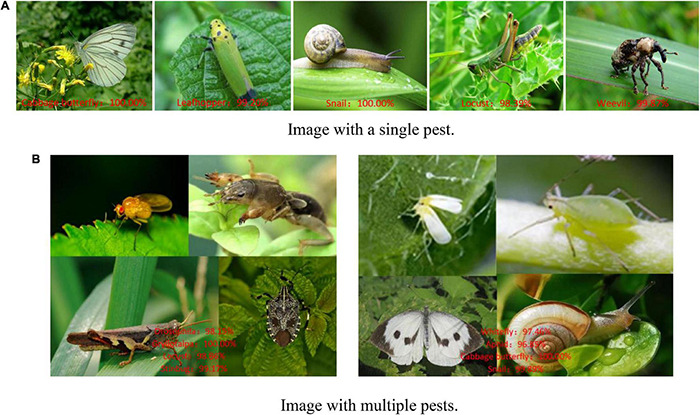
Recognition results for crop pests. **(A)** Image with a single pest. **(B)** Image with multiple pests.

Figure 9 shows the correct recognition results for randomly selected images using the ResNet-50-PCSA model. The model has a better recognition result of the 5 pest images in Figure 9A, and the accuracies of cabbage butterflies and snails are 100.00%. The accuracy of locust is the lowest, but it is also as high as 98.39%, which meets the accuracy requirements in real pest recognition tasks. In Figure 9B, we stitch images of different pests into one image and input it into the model. The model also obtained a better performance and could accurately recognize each pest in the stitched image.

**FIGURE 10:**
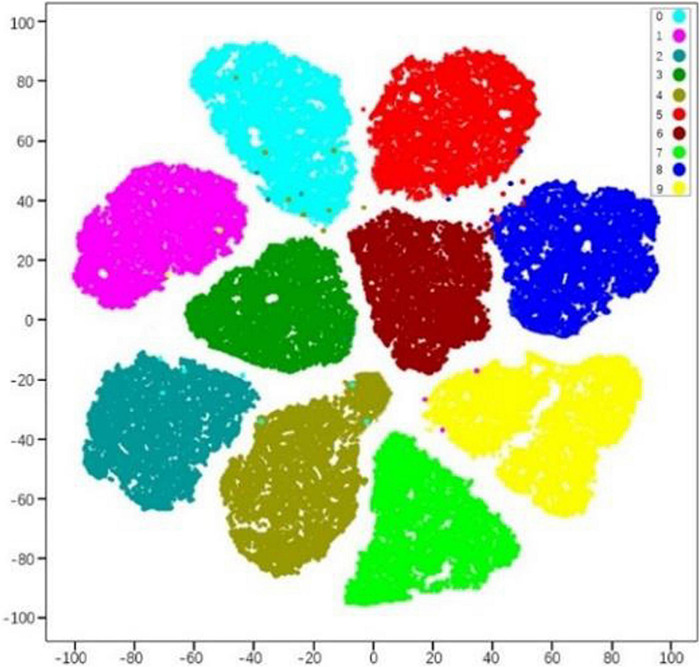
Clustering results of the training set.

To better show the classification performance of the model on 10 classes of pests, we choose t-SNE clustering for feature spatial distribution representation. The experiment extracts the features of each image from the fully connected layer of the ResNet-50-PCSA model, uses the t-SNE algorithm to visualize the high-dimensional features in a two-dimensional space of 10-class pests, and performs hierarchical clustering analysis on the features. The 2048-dimensional feature clustering results are shown in Figure 10. Each color represents the category of different pests, for a total of 10 categories. On the whole, the features reflected by different pests show a better clustering effect, which is the key to accurately distinguishing different pests. The distribution position of the feature clusters of the same variety deviates, mainly because in the real agricultural environment, the color and shape features of some pests are similar.

Based on the above results, it can be seen that the ResNet-50-PCSA model can complete the task of crop pest recognition well and has a high robustness and accuracy. This model can be a useful detection tool in the field of crop diseases.

### ResNet-50-PCSA Adaptability on Other Datasets

**FIGURE 11:**
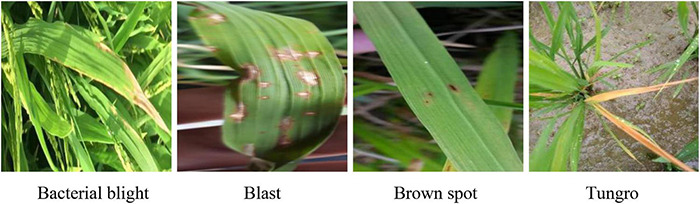
Sample images of the rice leaf dataset.

To further validate the practical application performance of our model, we experiment with the proposed method on other public datasets of rice leaf diseases, and the disease images have real agricultural backgrounds. The dataset contains 5,932 rice leaf disease images, which include bacterial blight, blast, brown spot and tungro. All the patches were treated as data samples and resized to *224* × *224* pixels, and Figure 11 shows the four varieties of rice leaf diseases.

Under the same training environments, GoogLeNet, ResNet-50 and Xception were selected for comparative experiments on rice leaf diseases. As shown in Table 7, the proposed model in this paper has an average detection accuracy of 99.35% for the 4 classes of rice leaf diseases. Compared with the GoogLeNet, Xception and ResNet-50 models, the accuracy is 5.67, 5.82, and 4.54% higher, respectively. The ResNet-50-PCSA model has the fastest average detection time for a rice leaf image, and the average detection time for a single rice disease image is only 0.85 ms slower than ResNet-50.

**TABLE 7:** The evaluation result of the rice leaf dataset.

Model	Input	P	R	F_1_	T_*a*_ (ms)	Accuracy (%)
GoogLeNet	256	0.9365	0.9361	0.9363	33.07	93.68
Xception	256	0.9355	0.9353	0.9354	31.51	93.53
ResNet-50	256	0.9480	0.9478	0.9479	30.54	94.81
ResNet-50-PCSA	256	0.9933	0.9935	0.9934	31.39	99.35

**FIGURE 12:**
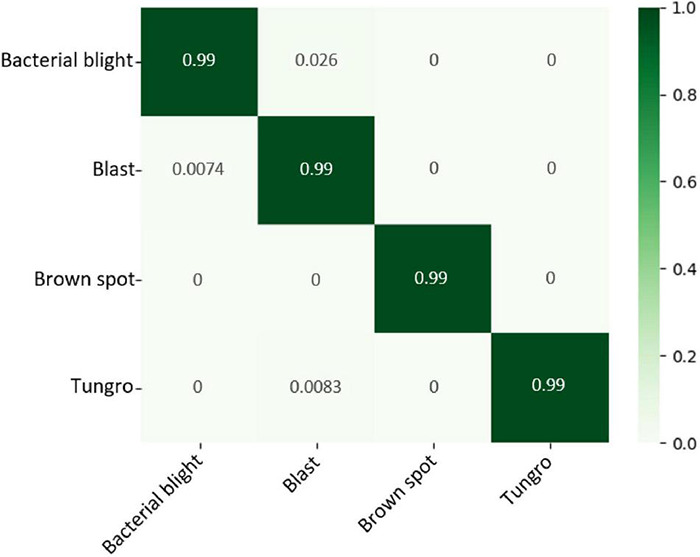
Confusion matrices for rice leaf diseases.

The detection result is represented by the confusion matrix in Figure 12, and the detection accuracy of 4 classes of rice leaf diseases exceeded 99%. Compared with crop pest recognition, the accuracy of rice leaf disease diagnosis has increased by 1.18%. The main reason is that there are only 4-classes of rice leaf diseases, which is 6-classes less than that of pest recognition. It is proven that the proposed method has a wide range of applicability and has better performance relative to deep-based methods on public datasets. Moreover, it is certified that our method is effective for datasets captured in real agricultural environments.

## Conclusion

In this work, a pest recognition model based on deep learning was proposed using a manually collected dataset to classify 10 types of crop pests. A total of 5,245 images were downloaded from different websites and manually validated. In the data preparation phase, data augmentation was used to expand the dataset. We successfully designed a parallel attention mechanism and deeply integrated the original ResNet-50 model and recognize the great performance of the proposed network through various experiments. The added attention module can suppress complex backgrounds and extract multiscale pest features more accurately without increasing the number of model parameters. Under the condition of ensuring high accuracy, rapid recognition is realized on images with multiple pests and complex backgrounds. It is verified that our method is of great significance and provides accessible help for the recognition of crop pests.

In this feature, we will use the proposed method to implement a crop pest image recognition system and transplant it into agricultural inspection robots. At the same time, we will also expand a dataset of crop pests in a real agricultural environment to improve the model performance of the robot. It can help farmers accurately distinguish pests, carry out pesticide works according to the types of pests, and successfully realize agricultural modernization and intelligence.

## Data Availability Statement

The original contributions presented in the study are included in the article/supplementary material, further inquiries can be directed to the corresponding author/s.

## Author Contributions

SZ and JL designed the study, performed the experiments, data analysis, and wrote the manuscript. SZ advised on the design of the model and analyzed to find the best method for improve detection accuracy of crop pest. YJ, ZB, and CH collected the data from the website and contributed the information for the data annotation. All authors contributed to the article and approved the submitted version.

## Conflict of Interest

The authors declare that the research was conducted in the absence of any commercial or financial relationships that could be construed as a potential conflict of interest.

## Publisher’s Note

All claims expressed in this article are solely those of the authors and do not necessarily represent those of their affiliated organizations, or those of the publisher, the editors and the reviewers. Any product that may be evaluated in this article, or claim that may be made by its manufacturer, is not guaranteed or endorsed by the publisher.

## Footnotes

1
https://www.kaggle.com/

2
https://www.forestryimages.org/index.cfm
